# Imaging Biomarkers in Radiotherapy

**DOI:** 10.3390/cancers18081232

**Published:** 2026-04-14

**Authors:** Dandan Zheng, Issam El Naqa, X. Sharon Qi, Anil Sethi, Filippo Alongi

**Affiliations:** 1 Department of Radiation Oncology, University of Rochester, Rochester, NY 14642, USA; 2 Machine Learning & Radiation Oncology, Moffitt Cancer Center, Tampa, FL 33612, USA; 3 Department of Radiation Oncology, University of California Los Angeles, Los Angeles, CA 90095, USA; 4 Department of Radiation Oncology, Loyola University Medical Center, Maywood, IL 60153, USA; 5 Department of Radiation Oncology, IRCCS Sacro Cuore Don Calabria Hospital, Negrar, 37024 Verona, Italy; 6 Department of Radiation Oncology, University of Brescia, 25121 Brescia, Italy

**Keywords:** biomarker, radiomics, imaging, radiotherapy

## Abstract

Advances in medical imaging modalities such as multiparametric and functional MRI, PET, and CT/CBCT, together with complementary innovations such as radiomics, artificial intelligence (AI), adaptive radiotherapy, and theranostics, have expanded the role of imaging from anatomical guidance to biologically informed treatment planning, adaptation, and response assessment. In this review, we provide a comprehensive overview of the technical foundations of imaging biomarkers in radiotherapy (RT), spanning functional and molecular imaging techniques and data-driven analytic approaches. We synthesize current clinical evidence across major disease sites, highlighting how imaging biomarkers are being used to refine target delineation, guide dose painting and functional avoidance, predict and monitor treatment response, and support adaptive and personalized RT strategies. We also critically examine key challenges to clinical translation and implementation, including standardization and reproducibility, validation and generalizability, interpretability of AI-driven models, regulatory and ethical considerations, issues of data sharing, reimbursement, and equity. Finally, we propose a multi-stage translational roadmap to guide the development, validation, and clinical deployment of imaging biomarkers in radiotherapy. Collectively, this review underscores the central role of imaging biomarkers in advancing biologically adaptive and precision radiotherapy and outlines priorities for their responsible and equitable integration into routine clinical practice.

## Simple Summary

Cancer radiotherapy has traditionally used standard medical images to see where a tumor is located. However, tumors are biologically complex and vary between patients and subregions of the tumor. This review explores using advanced medical images as biological markers. These biomarkers act like detailed maps that reveal a tumor’s inner characteristics, such as which areas lack oxygen or are growing most aggressively. The review also discusses how computer programs can find hidden patterns in these images to help doctors predict how a patient will respond to therapy. By using these detailed digital maps, medical teams can personalize radiation, delivering higher doses to difficult spots while better protecting the surrounding healthy organs, as well as adjusting treatments as the tumor changes. The review also presents current challenges and a future roadmap for imaging biomarkers to help move us toward a future where cancer care is safer, more effective, and tailored to each individual, ultimately improving survival rates and quality of life for patients.

## 1. Introduction

### 1.1. Definition of Biomarker and Imaging Biomarker

The application of high-throughput genomic, transcriptomic, and proteomic technologies has led to the discovery of molecular biomarkers in cancer [1]. These are molecular-level probes that characterize tumors and provide quantifiable insights to support a range of clinical applications, from early disease detection and diagnosis to treatment stratification and outcome prediction. In parallel, advances in medical imaging, including improved spatial and contrast resolution, digitization, the ability to image specific biological features or processes, and integration with molecular probes, have generated large volumes of imaging data [2]. These developments have uncovered a new class of biomarkers: imaging biomarkers. Imaging biomarkers are defined by the FDA-NIH Biomarker Working Group as objectively measured characteristics derived from in vivo images that reflect normal biological processes, pathogenic processes, or responses to therapeutic interventions [3]. Derived directly from medical images, imaging biomarkers capture macro-level phenotypes and spatial characteristics of normal tissues and tumors. They reflect both anatomical structure and functional or molecular properties, depending on the imaging modality and technique. In oncology, imaging biomarkers have been used to noninvasively assess tumor burden, heterogeneity, perfusion, metabolism, hypoxia, cellularity, etc. [4]. These developments are enabling imaging biomarkers’ application in disease diagnosis, prognosis, treatment response monitoring, and image-guided interventions [5]. The field continues to grow rapidly, offering a complementary and scalable approach to molecular profiling for personalized and precision cancer care [6]. In the context of radiotherapy (RT), imaging biomarkers encompass quantitative or qualitative features extracted from routine or advanced imaging that provide biologically relevant information beyond anatomy alone [7].

Imaging biomarkers may be broadly categorized according to the type of information they encode either as functional (e.g., diffusion, perfusion, ventilation) or molecular (e.g., PET tracer uptake reflecting metabolism, hypoxia, or receptor expression) [8]. From a clinical perspective, these biomarkers may further be classified based on their intended role as

- prognostic biomarkers: inform overall outcome independent of treatment;
- predictive biomarkers: identify the likelihood of benefit from a specific therapy;
- response biomarkers: assess treatment effect over time;
- surrogate biomarkers: serve as substitutes for clinical endpoints.

### 1.2. Creation and Clinical Applications of Imaging Biomarkers in RT

**Figure 1:**
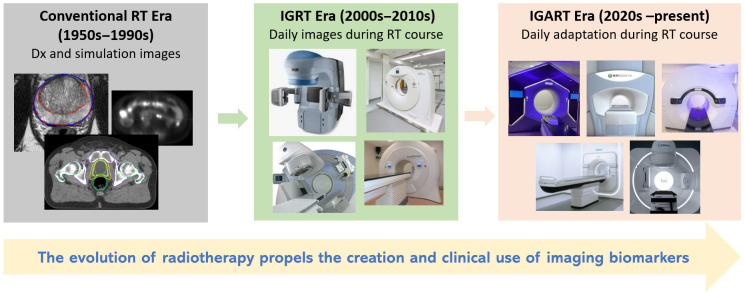
Evolution of radiotherapy (RT) and the growing role of imaging biomarkers. The progression from conventional RT (1950s–1990s) to image guided RT (IGRT) (2000s–2010s) and image guided adaptive RT (IGART) (2020s–present) reflects an expanding use of imaging data, from static anatomical references to longitudinal, dynamic, and biologically informed adaptation of treatment.

Imaging plays a central role in RT, serving as the foundation for designing treatment plans tailored to each patient’s anatomy and for providing verification and guidance to ensure accurate dose delivery. The evolution of RT has propelled the creation and clinical use of imaging biomarkers [9,10]. As illustrated in Figure 1, in the conventional RT era, simulation and diagnostic images primarily provided static anatomical information. In the image-guided RT (IGRT) era, the acquisition of daily images during treatment, especially volumetric images, has enabled longitudinal and dynamic assessment of anatomical and physiological/functional changes. In the image-guided adaptive RT (IGART) era, such information can now be directly leveraged to adapt RT based on evolving anatomy, physiology, and tumor biology. While many imaging biomarkers have traditionally been derived from a single pre-treatment imaging time point, increasing emphasis has been placed on longitudinal and on-treatment biomarkers, enabled by advances in image acquisition, IGART platforms, and computational analysis [11]. This evolution has expanded the role of imaging biomarkers from static risk stratification toward dynamic treatment adaptation, forming the foundation for biologically guided and personalized radiotherapy.

**Figure 2:**
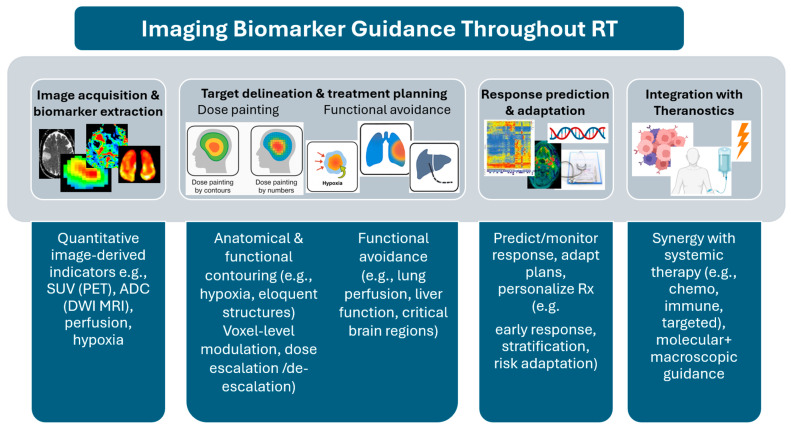
Imaging biomarkers can provide quantitative biological guidance across the entire RT workflow. From target delineation and dose painting to response prediction and integration with theranostics, these biomarkers support biologically informed, personalized RT strategies.

As quantifiable, image-derived indicators, imaging biomarkers can support and enhance various steps in the RT process. Figure 2 illustrates key applications of imaging biomarkers in RT.

Imaging biomarkers may be used in target volume delineation, ranging from anatomical tumor delineation in sites such as the brain, lung, and prostate, to functional and biological characterization, including hypoxia mapping and identification of eloquent or radiosensitive structures in the brain, lung, and abdomen [12]. These biomarkers enable the integration of biologically informed subvolumes into treatment planning.

In RT planning, imaging biomarkers support strategies such as dose painting for escalation in aggressive tumor regions and de-escalation in lower-risk areas, with the goal of improving tumor control while minimizing normal tissue toxicity [13]. They are also used in functional avoidance RT planning, where high-function regions (e.g., well-perfused lung, active parts of liver, and critical subregions of the brain) are selectively spared. To emphasize their quantitative potential, novel approaches have emerged that allow “paint by numbers”, applying voxel-level dose modulation based on imaging parameter values, compared to the traditional “paint by contours” approach [13].

Beyond planning, imaging biomarkers are also used to predict and monitor treatment response of both tumor and normal tissue during the course of RT. This supports initial treatment selection, informs treatment regimen stratification, enables risk-adapted plan modifications during the course of RT, and guides personalized prescription adjustments based on early response indicators.

Additionally, the emergence of theranostics opens new possibilities for synergy between RT and systemic therapies, including chemotherapy, immunotherapy, and targeted agents. By leveraging molecular-level and macroscopic guidance, these approaches enable a more comprehensive and biologically integrated strategy for personalized cancer treatment [14].

### 1.3. Rationale of the Review

Imaging biomarkers represent a critical and rapidly evolving frontier in radiation oncology. Their potential to provide noninvasive, quantitative insight into tumor and normal tissue characteristics could usher us into the next critical RT paradigm, transforming how we approach RT from target delineation and standard-of-care planning to personalized and biology-guided treatment adaptation and response assessment [15]. Imaging biomarkers can harness the advantages of several recent developments, including advanced imaging modalities, functional and molecular imaging, radiomics, artificial intelligence (AI), and modern online adaptive radiotherapy. These advances are poised to collectively push RT toward a more personalized, biology-informed paradigm.

Despite growing research interest and promising early applications, the integration of imaging biomarkers into routine radiotherapy practice remains in its early stages. While individual studies have demonstrated technical feasibility and clinical relevance, broader clinical adoption is still limited [16]. The existing literature is often fragmented, with review reports focused on specific modalities, disease sites, or isolated technical advances, and largely without a comprehensive synthesis that connects these developments across the RT workflow. There is a need to consolidate recent progress, clarify the clinical and technical foundations, and identify key opportunities and challenges that remain.

Therefore, this review aims to address that gap, with the content organized as follows. Section 2 will introduce the technical basis of imaging biomarkers, focusing on advances in imaging modalities, including magnetic resonance imaging (MRI), positron emission tomography (PET), and computed tomography (CT)/cone-beam computed tomography (CBCT). Section 3 will outline complementary innovations that support the development and application of imaging biomarkers, including radiomics, AI, online adaptive radiotherapy platforms, and theranostics. Section 4 will summarize current clinical evidence, organized by major disease sites, highlighting translational progress. Section 5 will discuss current challenges and emerging trends in the use of imaging biomarkers, with attention to issues such as standardization, validation, regulatory and ethical considerations, and equitable access. Finally, Section 6 will provide conclusions highlighting the potential and priorities for integrating imaging biomarkers into personalized, precision RT.

Given the rapid evolution and inherent heterogeneity of imaging biomarkers in radiotherapy, ranging from early-phase PET tracer studies to retrospective radiomic cohorts, this work adopts a narrative review methodology rather than a systematic review or meta-analysis. This approach is intentionally selected to provide a broad, integrative synthesis across diverse disease sites and modalities, allowing for a conceptual framework that contextualizes fragmented technical advances within the holistic radiotherapy workflow. While this narrative format allows for a more comprehensive overview of the translational landscape, we acknowledge the inherent limitations of this methodology, including potential selection bias and the absence of formal quantitative evidence synthesis common to systematic approaches.

## 2. Technical Basis—Advanced Imaging Modalities

Modern advances in medical imaging such as improvements in spatial and contrast resolution, the transition to digital imaging, and the development of modalities that can capture functional, physiological, and molecular processes have generated large volumes of data with potential for biomarker signals [17]. The integration of molecular probes and specialized imaging sequences further enhances this potential, offering insight beyond structural anatomy. This section introduces the key imaging modalities, multiparametric MRI, PET, and CT/CBCT, that serve as foundational platforms for deriving imaging biomarkers in RT.

### 2.1. MRI

MRI offers superior soft-tissue contrast and a suite of functional sequences, making it invaluable for RT planning in many cancers. Multiparametric MRI (mpMRI) combines anatomical imaging with functional techniques to provide a comprehensive assessment of tumor biology and tissue characteristics, offering rich image information for imaging biomarkers. mpMRI has played an important role in the RT of prostate, brain, and head and neck cancers [18,19].

#### 2.1.1. Diffusion-Weighted Imaging (DWI) and Diffusion Tensor Imaging (DTI) MRI

DWI and DTI are MRI techniques that probe the mobility and directional movement of water molecules within tissues. DWI quantifies the apparent diffusion coefficient (ADC), which is sensitive to cellular density and has been widely investigated as an imaging biomarker for tumor characterization and early treatment response [20]. DTI extends this by measuring anisotropic diffusion, enabling visualization of white matter tracts and spatial orientation of tissue microstructure, such as identifying and preserving critical neural pathways in brain RT. DWI and DTI biomarkers have been studied for functional avoidance RT in the brain and treatment response prediction and dose escalation in head and neck RT [21,22].

#### 2.1.2. Perfusion-Weighted Imaging (PWI) and Dynamic Contrast-Enhanced (DCE) MRI

PWI and DCE-MRI assess tissue perfusion and vascular characteristics by tracking the passage of contrast agents through the microvasculature. These techniques provide quantitative parameters such as blood volume, flow, permeability, and vessel leakiness, offering insight into tumor angiogenesis and hypoxia. In RT, these metrics can serve as signals to differentiate between recurrence and treatment response, identify aggressive or resistant tumor subregions, guide dose escalation, and monitor treatment response. Studies have shown potential in head and neck, brain, and cervical cancers [1,23].

#### 2.1.3. Magnetic Resonance Spectroscopic Imaging (MRSI)

MRSI extends conventional MRI by capturing the spatial distribution of metabolites within tissues to gain insight into tumor biochemistry. Key metabolites such as choline and creatine can be quantified to assess cellular proliferation, energy metabolism, and neuronal integrity. As an imaging biomarker, MRSI has been used to improve tumor delineation, identify infiltrative margins not visible on conventional imaging, and define biologically aggressive subregions for RT dose escalation, particularly in gliomas [24,25,26,27].

#### 2.1.4. Other Functional MRI (fMRI) Techniques

Beyond diffusion, perfusion, and spectroscopy, other fMRI techniques such as blood oxygenation level-dependent (BOLD) imaging and oxygen-enhanced MRI provide additional physiological insights relevant to radiotherapy. BOLD-fMRI, commonly used to map neural activity by detecting changes in deoxyhemoglobin, can help localize eloquent brain regions for functional avoidance RT [28]. Oxygen-enhanced MRI and tissue oxygen level-dependent (TOLD) imaging are also emerging tools to assess tumor oxygenation status, offering potential biomarkers of hypoxia [29,30]. Although still largely investigational in RT, these techniques show promise for guiding hypoxia-targeted dose escalation or combined modality therapy.

### 2.2. PET

PET provides molecular-level imaging by detecting radiolabeled tracers that target specific biological processes. In RT, PET plays a critical role in staging, target delineation, biological subvolume identification, and response monitoring. Depending on the tracer used, PET can visualize tumor metabolism, hypoxia, proliferation, receptor expression, etc. [31,32,33]. The following subsections highlight several key PET tracers with established or emerging relevance as imaging biomarkers in RT.

#### 2.2.1. ^18^F-Fluorodeoxyglucose (FDG) PET

^18^F-FDG PET, which images glucose metabolism, remains the most widely used PET imaging modality for cancer. It aids in staging, treatment planning, and target volume delineation, especially in head and neck and lung cancers [34,35,36]. FDG uptake is commonly used to define biological target volumes (BTVs) for dose escalation or de-escalation [37,38]. Quantitative measures such as maximum standardized uptake value (SUVmax), metabolic tumor volume, and total lesion glycolysis have been explored as prognostic biomarkers and for adaptive response assessment [39,40].

#### 2.2.2. Hypoxia PET

There are a few PET tracers that target tumor hypoxia, such as ^18^F-fluoromisonidazole (FMISO), ^18^F-HX4, and ^64^Cu-ATSM [41,42]. PET with these tracers provides spatial maps of oxygen-deficient regions that are often more radioresistant. These imaging biomarkers are being investigated for guiding hypoxia-targeted dose painting, stratifying treatment intensification, and selecting patients for hypoxia-modifying therapies [43,44]. Although not yet routine in clinical practice, early studies demonstrate feasibility and suggest potential benefit in tumors such as head and neck, lung, and cervical cancers [32,45]. While hypoxia imaging has clear prognostic and adaptive potential, routine clinical adoption remains early and largely investigational. Despite their prognostic value, the adoption of tracers like ^18^F-FMISO is currently hindered by limited accessibility, short half-lives requiring on-site cyclotrons, and complex pharmacokinetic modeling. The application is therefore limited to academic centers. Future adoption may rely on the development of more stable and more accessible tracers or the use of functional MRI surrogates (like BOLD or TOLD) to bring hypoxia mapping into broader clinical practice. Additional trials and multi-center feasibility studies are also crucial to determine whether the use of hypoxia imaging becomes essential for dose painting or therapy intensification.

#### 2.2.3. Prostate-Specific Membrane Antigen (PSMA) PET

PSMA PET, using tracers like ^68^Ga-PSMA, enables high-sensitivity detection of prostate cancer, including recurrence and micrometastatic disease. In the RT setting, PSMA PET improves staging and guides personalized treatment planning on PET-positive lymph nodes or oligometastases [46,47,48]. PSMA PET has proven useful in the early metastasis detection, treatment management, and guiding focal therapy [48]. It also serves as both a diagnostic and potential theranostic imaging biomarker, increasingly influencing salvage and definitive RT decisions in prostate cancer [49,50,51,52].

#### 2.2.4. Amino Acid PET

Amino acid PET uses tracers like ^11^C-methionine, ^18^F-fluoroethyltyrosine (FET), and ^18^F-DOPA [53,54]. Reflecting amino acid transport and protein synthesis, they offer improved tumor-to-background contrast in the brain compared to FDG-PET. They can serve as imaging biomarkers for tumor metabolism and proliferation. In RT, amino acid PET has been used to refine target delineation in gliomas, detect tumor infiltration beyond conventional MRI boundaries, and identify subregions for dose escalation [55]. They are also under investigation for distinguishing tumor recurrence from radiation necrosis during post-RT follow-up [55].

### 2.3. CT and CBCT

CT and CBCT imaging are integral to RT planning and delivery, providing essential anatomical detail and geometric accuracy. While traditionally used for structure-based planning, recent advances in spectral imaging, temporal resolution, and image quality have enabled the extraction of quantitative metrics and functional information [56,57]. These developments expand the role of CT-based techniques beyond anatomical visualization, positioning them as potential sources of imaging biomarkers for functional mapping, response assessment, and adaptive RT.

#### 2.3.1. Dual-Energy CT (DECT) and Photon-Counting CT (PCCT)

DECT has become increasingly prevalent in RT departments, especially those with proton and heavy particle therapies. PCCT is currently in the early stages of clinical applications and is mostly confined to the academic diagnostic departments. DECT acquires imaging data at two different energy levels, enabling material decomposition and quantitative tissue characterization [58,59,60]. These techniques generate virtual monoenergetic images, iodine concentration, and effective atomic number maps, which can enhance contrast resolution and tissue differentiation. PCCT is an emerging technology that uses advanced photon-counting detectors to enable ultra-high-resolution and multi-energy CT imaging with improved tissue contrast and noise reduction [61]. In RT, DECT-derived biomarkers such as iodine perfusion maps have been explored for functional lung imaging, tumor characterization, and improving dose calculation accuracy, particularly in proton therapy [60]. It is also being investigated to guide functional avoidance strategies and to identify tumor subregions with distinct vascular properties. As a next-generation spectral imaging modality, PCCT offers enhanced spatial resolution, material decomposition, and quantitative accuracy, holding promise for the development of imaging biomarkers related to tissue composition, tumor heterogeneity, and functional characterization in RT.

#### 2.3.2. Four-Dimensional CT (4DCT) and Functional Lung Imaging

4DCT is widely used for thoracic and abdominal RT. It captures respiratory motion, providing phase-resolved volumetric images. Beyond motion management and internal target volume generation, 4DCT data can be processed to derive ventilation maps using density-change or deformable image registration-based methods. These ventilation maps offer a surrogate measure of regional lung function and have been proposed as imaging biomarkers to guide functional lung avoidance in thoracic RT [62,63]. Although variability and reproducibility remain challenges, 4DCT-based functional imaging is gaining traction as a practical, noninvasive tool for personalizing lung dose distributions.

#### 2.3.3. CBCT

CBCT is routinely used for daily patient setup and image guidance in RT. Traditionally limited by lower image quality, recent software and hardware advancements have significantly improved its soft-tissue contrast and quantitative consistency. Enhanced CBCT opens opportunities for extracting radiomic and delta-radiomic features as imaging biomarkers. Systems like HyperSight (Varian Medical Systems, Palo Alto, CA, USA) offer fast acquisition, improved spatial resolution, and noise reduction [64]. Such systems are currently investigated for diffusion and perfusion imaging, making CBCT increasingly viable not just for positioning but also for anatomical and functional monitoring during RT treatment [65].

**Figure 3:**
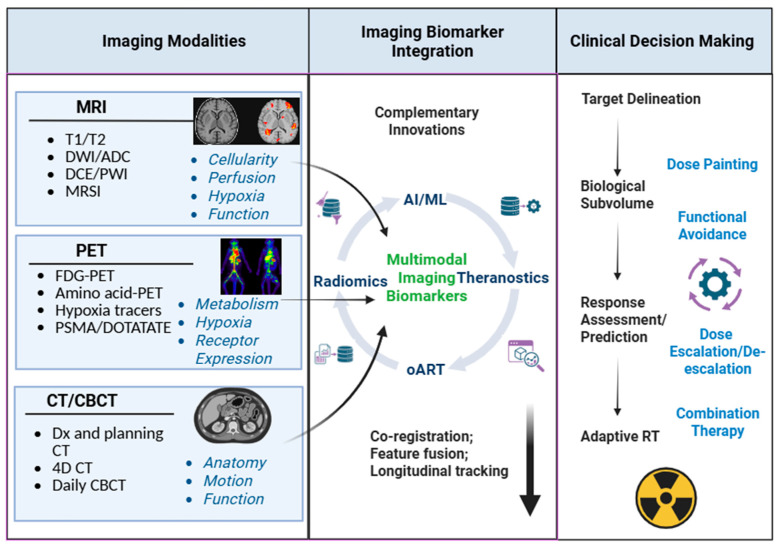
Conceptual framework for integrating imaging biomarkers from different modalities, complementary innovations, and clinical decision-making in biologically guided radiotherapy. Dx: diagnosis. For other abbreviations, please refer to the “list of abbreviations” at the end of the paper.

While individual imaging modalities provide valuable but partial views of tumor anatomy and biology, their greatest clinical impact emerges through integration across modalities and over time. Modern radiotherapy increasingly relies on complementary innovations detailed in the next section to extract, fuse, and interpret high-dimensional imaging information and translate it into actionable clinical insight. Figure 3 illustrates a conceptual framework linking imaging modalities to complementary innovations and downstream clinical decision-making. In this framework, MRI, PET, and CT/CBCT contribute distinct yet overlapping biological information that is integrated into multimodal imaging biomarkers through feature fusion, co-registration, and longitudinal tracking. These integrated biomarkers form the basis for biologically informed target definition, response assessment and prediction, and adaptive treatment strategies, serving as the foundation for personalized and biologically adaptive RT.

## 3. Technical Basis—Complementary Innovations

In addition to advanced imaging modalities, several complementary innovations are accelerating the development and clinical utility of imaging biomarkers in RT. These include data-driven approaches such as radiomics and AI, the emergence of online adaptive radiotherapy (oART) platforms that enable frequent on-treatment imaging, and the growing field of theranostics that integrates diagnostic and therapeutic functions. Together, these innovations enhance the extraction, interpretation, and clinical application of quantitative imaging data to support personalized and biologically adaptive treatment strategies. Importantly, they serve as integration and deployment layers that enable multimodal, longitudinal imaging biomarkers to inform real-time clinical decision-making.

### 3.1. Radiomics

The high-throughput extraction of quantitative features from medical images, termed radiomics, has become a rapidly growing field over the past decade and a half [2,7,66,67]. Traditional (handcrafted) radiomics derives features such as intensity, shape, texture and high-order features to characterize tumor phenotype and heterogeneity. Deep learning (DL)-based radiomics automatically learns hierarchical imaging features directly from data without predefined feature engineering [68]. Radiomic features may be derived from single or multiple imaging modalities and increasingly from longitudinal datasets, enabling the characterization of temporal heterogeneity through approaches such as delta-radiomics [69,70,71,72,73]. While static radiomics involves the extraction of features from a single-time point image (typically pre-treatment) to provide prognostic risk stratification, delta-radiomics quantifies the change in these features over time [11]. By analyzing longitudinal variations during or after treatment, delta-radiomics captures the dynamic biological response of the tumor and normal tissues. This temporal information is particularly vital for response assessment, as changes in texture or heterogeneity often precede macroscopic anatomical changes, enabling earlier treatment adaptation [11,69,70,71]. As such, static and delta-radiomics serve as imaging biomarkers for detection, diagnosis, risk stratification, outcome prediction and treatment response assessment. Within the specific context of radiotherapy, “detection” represents a multi-dimensional clinical scope. This could include the identification of sub-clinical microscopic disease, the localization of distinct biological niches such as hypoxic or hypermetabolic zones, sensitive detection of recurrence, and the post-treatment differentiation between disease recurrence and radiation-induced effects. Altogether, radiomics is being explored in RT for target delineation, biologically guided dose painting, treatment adaptation, and more. Ongoing work focuses on validating robust, reproducible signatures and integrating radiomics into clinical decision-making.

### 3.2. AI

Over the past decade, there has been an explosion of development and applications of data-driven AI approaches, such as deep learning (DL), DL-based foundation model such as large language models (LLMs). These methods are increasingly applied to integrate high-dimensional imaging biomarkers across modalities and time points, enabling improved prediction of treatment response, toxicity, and survival beyond conventional single-modality or single–time point analyses. DL models can discover complex patterns from raw imaging data and are being developed to predict tumor control and treatment-related adverse effects. In parallel, LLMs and AI-assisted platforms are facilitating data interpretation, protocol standardization, and clinical reporting. Specifically, these tools are being utilized to automate the extraction of clinical metadata from unstructured electronic health records and to structure radiology reports into standardized templates [74]. Furthermore, LLMs can assist in the synthesis and semantic interpretation of high-dimensional radiomics features, bridging the gap between abstract mathematical descriptors and clinical biological phenotypes [75,76]. The synergy between AI and quantitative imaging biomarkers is accelerating their clinical translation; however, challenges related to generalizability, interpretability, explainability, and robust validation remain active and important areas of research [77,78].

### 3.3. oART Platforms for Frequent Longitudinal On-Treatment Imaging

oART platforms, such as MR linac, CBCT-guided systems, and the newest PET-guided platform, enable frequent, high-quality on-treatment imaging that supports online adaptation of the initial RT treatment plan based on daily anatomical and potentially biological changes [79]. These platforms create unique opportunities to longitudinally monitor tumor response throughout the course of treatment. Coupled with various MRI sequences, PET tracers, and CBCT-based functional imaging or delta-radiomics, these platforms enable dynamic application of imaging biomarkers to guide plan adaptation and inform mid-treatment decision-making. By facilitating frequent on-treatment imaging, oART platforms support the transition from prognostic to response-driven and adaptive imaging biomarkers, supporting treatment modification based on observed biological and anatomical changes rather than relying solely on pre-treatment assumptions.

### 3.4. Theranostics

Theranostics refers to the combined use of diagnostic imaging and targeted therapy using the same or paired molecular agents [14,80]. In RT, theranostic agents such as PSMA or somatostatin-receptor-targeted compounds can be used to visualize tumor burden and simultaneously deliver therapeutic radionuclides [52]. These agents provide highly specific imaging biomarkers that inform treatment selection, track biological response, and potentially serve as adjuncts or alternatives to external beam RT. The integration of theranostics with RT planning and delivery is an emerging area of interest in personalized cancer treatment. As such, theranostic imaging biomarkers offer a direct link between tumor biology, treatment selection, and response assessment, complementing external beam radiotherapy within a unified precision oncology framework.

## 4. Clinical Evidence of Imaging Biomarkers

The clinical application of imaging biomarkers in radiotherapy has been explored across a wide range of cancer sites, each presenting unique anatomical and biological challenges. This section summarizes key investigations and clinical evidence from both prospective and retrospective studies, organized by disease site, to highlight how imaging biomarkers are being used to enhance target delineation, guide biologically adaptive planning, monitor treatment response, and personalize RT. Key examples from brain, lung, prostate, abdominal, and head and neck cancers are presented to illustrate the evolving translational impact of these technologies in practice.

### 4.1. Brain Tumors

Advanced imaging has become integral for guiding RT in treating primary and metastatic brain tumors. mpMRI provides superior soft-tissue contrast and functional detail in the context [81]. For example, DWI reveals tumor cellularity, while perfusion MRI maps tumor vascularity to distinguish viable tumor or recurrence from necrosis. Such MRI biomarkers have been used to aid target delineation and assess treatment responses beyond conventional MRI [82,83,84,85]. Furthermore, MRSI can detect metabolic aberrations in gliomas, helping define microscopic tumor extension. Incorporating MRSI into RT planning has been shown to alter target volumes and may reduce marginal recurrences [24,25,26]. Additionally, fMRI and DTI tractography are increasingly used to map critical cortex and white-matter tracts, enabling functional avoidance brain RT [86,87]. RT plans are designed to spare eloquent areas such as motor and language pathways without compromising tumor target coverage in these advanced RT approaches.

Amino acid PET imaging such as FET-PET offers high biological specificity for viable glial tumor tissue, making it particularly valuable for distinguishing tumor recurrence from radiation-induced changes and for prognostication [53,55]. Clinical studies have demonstrated that PET can substantially expand and refine target volumes in glioblastoma (GBM) [88,89]. For example, a planning study in GBM found FET-PET defined biological tumor volumes (BTV) were significantly larger than MRI-defined GTV (median 43.9 vs. 34.1 cc), with major geometric differences in 65% of cases [90]. PET/MRI fusion also reveals tumor infiltration beyond MRI contrast enhancement, prompting clinicians to cover PET-positive regions at risk. Beyond diagnostic and planning applications, amino acid PET–derived biomarkers have demonstrated robust prognostic value. A retrospective analysis of 146 GBM patients showed that a larger FET-PET metabolic volume at RT planning was an independent predictor of worse progression-free and overall survival [91]. This suggests these biomarkers can be used in risk stratification, where patients with large metabolic tumor burden might benefit from intensified or novel therapies. Amino acid PET has demonstrated particular utility in differentiating true tumor progression or recurrence from post-radiation treatment effects, including radionecrosis, where conventional MRI findings may be equivocal [92]. For example, amino acid PET can help differentiate true tumor progression from post-radiation changes, adding complementary information to MRI biomarkers. Early post-RT PET uptake reduction or an increase in ADC on DWI can indicate treatment response, whereas persistent high uptake or decreased diffusion might prompt investigation for residual disease. Similarly, ^68^Ga-DOTATATE PET and MRI are also combined to guide RT planning and response assessments for meningiomas [93].

Hand-crafted radiomic features extracted from MRI and PET scans have also been correlated with molecular subtype, treatment response, and survival in gliomas [31,94,95,96,97]. In patients with brain metastases, radiomic analysis of PET has shown utility in distinguishing radionecrosis from tumor recurrence. MRI-based perfusion metrics and diffusion-derived ADC values provide complementary information regarding vascularity and cellularity, respectively, and when interpreted alongside amino acid PET, can improve confidence in response assessment and disease characterization. Together, amino acid PET and advanced MRI biomarkers offer new insights into tumor biology, enabling improved target definition, prognostication, and post-treatment assessment in brain radiotherapy.

### 4.2. Head and Neck Cancer

Head and neck squamous cell carcinoma (HNSCC) represents a prototypical disease site for biology- and response-adaptive radiotherapy, where imaging biomarkers inform staging, risk stratification, treatment intensification or de-escalation, and longitudinal adaptation. Among available modalities, FDG PET and advanced MRI provide complementary biological and functional information that extends beyond conventional anatomical imaging.

FDG PET is widely used for staging primary tumors and nodal disease in head and neck cancer and plays a central role in RT target delineation [38,98]. PET improves detection of involved lymph nodes and reduces geographic miss, particularly in complex nodal basins. Beyond planning, FDG PET also provides prognostic and response information, with residual or persistent uptake after induction chemotherapy or early during chemoradiation associated with inferior locoregional control and survival.

Beyond glucose metabolism, hypoxia PET imaging has emerged as a biologically specific imaging biomarker in head and neck cancer, reflecting a well-established mechanism of radioresistance. Hypoxia-sensitive PET tracers such as ^18^F-fluoromisonidazole (FMISO) enable noninvasive identification and spatial mapping of hypoxic tumor subvolumes [44]. Clinical studies have shown that hypoxic tumors exhibit inferior local control with standard chemoradiation, whereas hypoxia-guided dose escalation to FMISO-avid regions can improve local control [43,99]. Meta-analyses of randomized trials have confirmed that modifying tumor hypoxia significantly improves locoregional control and overall survival, establishing hypoxia as one of the most clinically relevant biological targets in radiation oncology [100]. Biological modeling suggests that hypoxic dose painting can technically facilitate escalation up to 84 Gy, significantly increasing tumor control probability without exceeding normal tissue constraints [101]. A randomized phase II trial investigated FMISO-PET–guided dose escalation in locally advanced head and neck cancer [102], in which patients with PET-evident hypoxia were randomized to standard chemoradiation or escalated dose to hypoxic subvolumes. Although the study closed early, patients without significant hypoxia demonstrated superior 5-year local control compared with those with hypoxic tumors, confirming PET-defined hypoxia as a prognostic biomarker for local failure. Ongoing studies, including NRG-HN001, continue to evaluate hypoxia-guided treatment intensification using alternative tracers [103].

Advanced MRI techniques, particularly diffusion-weighted imaging, have also been investigated as early response imaging biomarkers in head and neck cancer. Prospective studies have demonstrated that an early increase in ADC during chemoradiation correlates with improved locoregional control, reflecting reduced tumor cellularity [104]. These findings support response-adaptive strategies, whereby patients with suboptimal ADC changes or persistent PET uptake may benefit from treatment intensification, while early responders could be considered for de-escalation [105]. Clinical trials such as ECOG-ACRIN 3311 and related studies are actively exploring response-based deintensification strategies in select populations, particularly HPV-positive oropharyngeal cancer, with encouraging early outcomes [106]. In head and neck cancer, multimodal imaging biomarkers, integrating PET- and MRI-derived features, are increasingly used alongside molecular biomarkers to improve risk stratification, response assessment, and adaptive treatment decision-making [40,107].

Adaptive radiotherapy is already widely implemented in head and neck cancer to account for significant anatomical changes during a 6–7 week treatment course, including tumor regression and weight loss [108,109,110]. Mid-treatment replanning has been shown to improve normal tissue sparing, particularly for salivary glands, and reduce late toxicity. With the advent of MR-guided and CT-guided radiotherapy, online adaptive planning is increasingly explored, enabling daily visualization of soft tissue changes and real-time contour adaptation. Early studies have demonstrated improved target coverage and reduced dose to organs at risk, although mature clinical outcome data are still pending [108].

In head and neck cancer, emerging molecular imaging targets illustrate the future potential of theranostic strategies that directly link diagnostic imaging biomarkers with targeted radionuclide therapies. One such target is the chemokine receptor CXCR4, which is overexpressed in a variety of solid tumors and can be imaged in vivo using CXCR4-directed PET tracers such as ^68^Ga-Pentixafor, facilitating noninvasive assessment of receptor expression and patient selection for potential targeted therapies. Prospective imaging studies using ^68^Ga-Pentixafor PET/CT have demonstrated CXCR4 expression in oral cavity, oropharyngeal, and nasopharyngeal squamous cell carcinomas, with uptake correlating to immunohistochemical staining, supporting its role as a candidate imaging biomarker for patient stratification [111]. Although less common, salivary gland malignancies such as adenoid cystic carcinoma also demonstrate PSMA expression detectable by PSMA-targeted PET, and small case series suggest the feasibility of PSMA radioligand therapy in this setting, reflecting a theranostic paradigm where imaging can identify suitable targets for subsequent therapy [52]. These investigational approaches exemplify how molecular imaging biomarkers may be coupled to targeted radionuclide therapies, expanding the translational landscape of imaging-guided personalized therapy in head and neck cancers.

### 4.3. Lung Cancer

Imaging has revolutionized radiotherapy planning in lung cancer, with ^18^F-FDG PET serving as the primary biological imaging biomarker, complemented by 4D-CT for motion characterization and functional assessment. ^18^F-FDG PET is now standard for staging and target delineation in non-small cell lung cancer (NSCLC). PET identifies metabolically active tumor regions and occult nodal metastases that CT alone might miss. Clinical evidence shows PET upstages a substantial fraction of patients and reduces geographic misses. For example, additional involved lymph nodes are detected in ~20% of cases, leading to enlargement of radiation fields in prospective series [47,50]. By fusing FDG PET with the planning CT, radiation oncologists can delineate more precise targets that encompass PET-avid disease while sparing atelectatic but non-tumoral lung tissue. FDG-PET is increasingly used as both a response and prognostic imaging biomarker, where decreases in mid-treatment standardized uptake value (SUV) correlate with pathologic response and survival, forming the biological basis for dose painting and adaptive radiotherapy strategies.

Dose painting and ART guided by functional imaging are active areas of research for lung cancer. The landmark adaptive trial NRG-RTOG1106 tested escalating radiation dose based on mid-treatment FDG-PET in locally advanced NSCLC [112]. This trial represents a landmark example of PET-defined response biomarkers directly informing adaptive dose escalation in lung radiotherapy. In this Phase II randomized study, patients in the experimental arm underwent a repeat PET after 40–50 Gy, with residual high-uptake subregions boosted to ~80 Gy total dose, while the standard arm received 60 Gy uniformly. The trial has demonstrated the feasibility and safety of PET-guided adaptive dose escalation for the first time in a multicenter setting. In-field tumor control appeared improved, with roughly 1% gain per additional Gy delivered, without significantly increasing toxicity. Although overall survival was not markedly different at interim analysis, likely due to sample size, this approach achieved the protocol-specified target for 2-year local control, warranting a Phase III investigation. Beyond FDG metabolism, hypoxia-targeted PET tracers such as ^18^F-FMISO provide complementary biological specificity, enabling identification of radioresistant subvolumes for biologically guided dose escalation [32,94,113]. Early-phase trials in Europe showed it is dosimetrically feasible to boost FMISO-avid subvolumes to >80 Gy, and retrospective analyses suggest patients with high baseline hypoxia PET uptake have poorer local control, reinforcing the rationale for biologic escalation [114].

In contrast to tumor-focused PET biomarkers, functional imaging of normal lung provides complementary information for toxicity mitigation and treatment planning. Rather than treating all lung tissues equally, imaging biomarkers can identify well-functioning lung regions to preferentially spare them, thus reducing toxicity such as pneumonitis. One such approach uses 4D-CT ventilation imaging to create patient-specific lung function maps [62]. In a recent multi-institutional Phase II trial, 4DCT ventilation was used to plan IMRT that avoided high doses to the most ventilated lung regions, meeting its primary endpoint of achieving a low Grade2+ pneumonitis rate at ~15% (10/67 patients) with functional avoidance, below the historical control threshold [63]. Patient-reported quality-of-life (QOL) outcomes were also favorable, with only ~30% of patients reported clinically significant decline in lung-related QOL at 1 year. Also, no statistically significant differences in patient-reported symptoms were found when correlating with functional dose metrics. These findings indicate that avoiding functional lung using imaging biomarkers can maintain pulmonary function and QOL after chemoradiation. Larger ongoing trials are evaluating if this translates into reduced symptomatic pneumonitis vs. conventional plans [115,116]. Other modalities like SPECT ventilation-perfusion and DECT with xenon contrast have similarly been used to guide lung-sparing treatments [117,118,119,120].

Beyond conventional PET metrics, radiomic features derived from PET and CT capture spatial heterogeneity and have demonstrated strong prognostic value in lung cancer, independent of clinical factors [66]. These types of findings were even validated in external cohorts, suggesting a generalizable imaging phenotype of tumor aggressiveness [121,122]. Such prognostic models could help identify patients who might benefit from therapy intensification or, conversely, those who could de-escalate treatment. Delta-radiomic analyses further extend single-time-point biomarkers by capturing temporal changes during treatment, offering additional prognostic and response information [70]. Notably, emerging biology-guided radiotherapy (BgRT) systems represent a direct extension of PET-based imaging biomarkers, using real-time PET emission to localize and treat biologically viable tumors during delivery [123,124]. While still in its infancy, early studies with BgRT in lung cancer have shown it can localize lesions during treatment and potentially allow smaller margins and asynchronous targeting of multiple lesions [124,125]. Together, PET-based tumor biomarkers and various functional lung imaging biomarkers provide guidance for biologically adaptive, toxicity-aware radiotherapy in lung cancer.

### 4.4. Prostate Cancer

Prostate cancer is a flagship disease site where mpMRI and PSMA PET serve complementary but distinct roles as imaging biomarkers, informing intraprostatic target definition and focal dose escalation on one hand, and sensitive staging, recurrence localization, and theranostic selection on the other. mpMRI with T2, DWI and dynamic contrast enhancement is now routinely integrated into RT planning for localized prostate cancer [126]. In addition to aiding standard target delineation, it can also localize the dominant intraprostatic lesion (DIL) for dose painting or boosting [66,127,128,129]. In this context, mpMRI functions as a spatially precise intraprostatic imaging biomarker, enabling selective intensification of radiation dose to biologically dominant disease. Clinical evidence supports boosting these MRI-defined tumor foci to higher radiation doses to prevent recurrence. The Phase III FLAME trial tested this by delivering a focal boost up to 95 Gy to MRI-visible lesions while treating the whole prostate to 77 Gy [130]. After 5 years, the boost arm showed significantly higher biochemical disease-free survival (92% vs. 85%, HR 0.45, *p* < 0.001) compared to standard treatment, while the toxicity was similar between arms. Results like this validate the role of mpMRI imaging biomarkers in guiding dose-escalation to dominant disease as a safe and effective strategy in intermediate- and high-risk prostate cancer. Similarly, additional studies such as the hypo-FLAME trial have extended this to ultrahypofractionated SBRT with an integrated boost, reporting similar improvements [131].

Beyond intraprostatic imaging, PSMA PET has emerged as the dominant molecular imaging biomarker for defining disease extent in prostate cancer, with major implications for nodal and metastatic staging. PSMA-targeted PET such as using ^68^Ga-PSMA-11 or ^18^F-DCFPyL has demonstrated much higher sensitivity than conventional imaging, directly impacting RT planning [132]. In a German study of patients planned for primary definitive RT, integrating ^68^Ga-PSMA PET led to staging changes in over half and altered the RT plan in a third of cases [47]. Specifically, PET often revealed unsuspected pelvic lymph node metastases, leading to additional nodal volumes being irradiated in a quarter of patients, and PET-positive nodes were given dose boosts in the majority of those cases. In a small fraction, PSMA PET even down-staged disease for negative nodes. As such, PSMA PET improves target volume delineation on microscopic nodal disease or distant oligometastases and can hence optimize RT treatment scope. These findings illustrate how PSMA PET–derived imaging biomarkers directly alter radiotherapy target volumes, dose prescription, and treatment intent. In the post-prostatectomy salvage setting, PSMA PET serves as a highly sensitive recurrence-localization biomarker at low PSA levels, enabling focal salvage radiotherapy with improved target confidence [48,133]. Clinical outcome data show that PSMA-PET-guided salvage RT yields high in-field tumor control rates for men with recurrent prostate cancer [46,133]. For instance, one report on PSMA-directed salvage RT noted excellent 3-year progression-free survival in patients whose treatment was adjusted based on PET findings [48]. These improvements in management have led guidelines to recommend PSMA PET for high-risk and recurrent prostate cases [134].

Beyond guiding external beam RT, PSMA imaging is pivotal in theranostics by pairing diagnostics with radionuclide therapy. The VISION trial selected patients with metastatic castration-resistant prostate cancer (mCRPC) based on PSMA PET and then randomized these patients to receive ^177^Lu-PSMA-617 radionuclide therapy [49]. The results showed that radiographic PFS was significantly prolonged (HR 0.40) and overall survival improved from 11.3 to 15.3 months (HR 0.62, *p* < 0.001) with PSMA-targeted radionuclide. This paradigm exemplifies the role of PSMA PET as both a predictive and selection biomarker, directly linking imaging phenotype to therapeutic benefit.

Radiomics and delta-radiomics further extend prostate imaging biomarkers by quantifying spatial heterogeneity and temporal changes on mpMRI and PSMA PET, offering additional prognostic and response information [51,71,135,136,137]. Emerging oART platforms, with improved image quality and frequent on-treatment imaging, provide a framework for longitudinal assessment and potential adaptive application of these biomarkers during radiotherapy.

Together, imaging biomarkers such as from mpMRI and PSMA PET provide complementary and synergistic insights for prostate radiotherapy, enabling biologically informed target definition, disease extent characterization, treatment intensification, and theranostic integration.

### 4.5. Abdominal and Gastrointestinal (GI) Cancers

In abdominal and GI malignancies, a diverse set of imaging biomarkers are applied in a site-specific manner, integrating anatomical, functional, and molecular information to guide radiotherapy planning, adaptation, and response assessment. In hepato-pancreato-biliary (HPB) cancers, contrast-enhanced multiphase CT and MRI are critical for mapping disease and are routinely used for RT planning and tumor delineation [138,139]. Functional and molecular imaging modalities, such as Tc-99m MAA SPECT and gadoxetate-enhanced MRI, are also used to inform selective radiation targeting and functional avoidance in liver cancer by characterizing regional hepatic function and perfusion [140,141]. In abdominal sites with poor tumor contrast and complex background such as pancreatic cancer, DECT has shown particular benefit in tumor delineation via generating virtual monoenergetic images and material density maps and enhancing tumor contrast [58]. Radiomics biomarkers have been investigated on these DECT images [142]. In this setting, DECT-derived imaging biomarkers primarily enhance target conspicuity rather than tumor biology, serving as an anatomical–functional complement to other modalities. For pancreatic cancer, daily visualization of changing anatomy and streamlined plan adaptation on oART platforms also allowed safe dose escalation while controlling toxicity to OARs like duodenum and stomach. The prospective single-arm “SMART” trial (MR-guided adaptive SBRT for pancreas) using MR linacs demonstrated high local control (~80–90% at 1 year) and low Grade3 GI toxicity (~5%), which compares favorably to historical cohorts [143,144]. This trial illustrates how frequent high-quality on-treatment imaging enables longitudinal assessment and safe dose escalation in anatomically complex abdominal sites.

mpMRI has also been explored and applied in the abdomen to enhance tumor contrast and to provide functional and molecular characteristics of the cancer [145]. In liver cancer, such imaging biomarker information has been utilized to guide functional avoidance RT [146,147]. Functional MRI sequences such as DWI provide response imaging biomarkers, where mid-treatment or post-neoadjuvant ADC changes correlate with pathological complete response (pCR) in rectal cancer. Prospective trials utilized MRI and endoscopic biomarkers to select suitable patients for less aggressive treatments, achieving organ preservation in a substantial subset without compromising survival [148]. These studies exemplify the use of imaging biomarkers to guide treatment de-escalation and organ preservation through response-adaptive decision-making. In esophageal cancer, FDG PET serves as the dominant molecular imaging biomarker for staging and identification of involved lymph nodes for radiotherapy planning. Clinical trials also stratified patients by PET response after induction chemotherapy to guide therapy modulation and demonstrated efficacy [149]. Similarly, in anal cancer, MRI and FDG PET–derived biomarkers are increasingly used as response imaging biomarkers, enabling mid-course assessment and adaptive dose modification such as boosting residual metabolically active disease [150,151].

Radiomics analysis and deep learning–based AI models provide a unifying framework across abdominal and GI cancers, enabling extraction of prognostic and response imaging biomarkers from pre-treatment, on-treatment, and post-treatment images across pancreatic, liver, rectal, and other GI malignancies [152,153,154,155,156,157]. These imaging-derived biomarkers have also been linked to underlying biological phenotypes and molecular characteristics, reinforcing their role as noninvasive surrogates of tumor biology [158,159,160]. On the theranostics front, molecular imaging biomarkers play a direct role in patient selection and treatment guidance. For example, ^68^Ga-DOTATATE PET imaging identifies somatostatin receptor–positive disease and guides the application of ^177^Lu-DOTATATE peptide receptor radionuclide therapy [161,162]. The NETTER-1 trial showed that this approach doubled progression-free survival in metastatic NETs for these imaging biomarker-selected patients [163]. For primary liver cancer, ^99m^Tc-MAA SPECT is used before radioembolization therapy to predict and optimize ^90^Y microsphere deposition and to avoid lung shunt [164]. These examples highlight the strongest form of imaging biomarker integration, where molecular imaging directly determines therapeutic eligibility and delivery.

Collectively, abdominal and GI cancers illustrate how anatomical, functional, and molecular imaging biomarkers, deployed longitudinally and supported by adaptive platforms, enable site-specific, biology-informed radiotherapy strategies.

### 4.6. Overall Clinical Evidence

Although the clinical impact of imaging biomarker-guided radiotherapy is increasingly recognized, current evidence remains early and often limited to isolated or single-institution studies. Table 1 summarizes some representative examples from this section. These examples illustrate the transition from geometric targeting to biologically informed strategies, showing improved oncologic control or reduced normal tissue toxicity across diverse disease sites. The current scarcity of large, randomized trials motivates the need for more systematic development and validation pathways, as outlined in the roadmap introduced in the next section.

## 5. Challenges, Trends, and a Roadmap

Despite rapid advances in imaging technologies and analytical methods, the clinical translation of imaging biomarkers in radiotherapy still faces many challenges across multiple domains.

### 5.1. Standardization and Reproducibility

A key barrier is the lack of standardized protocols for image acquisition, reconstruction, preprocessing, and feature extraction across institutions, vendors, modalities, and investigators. Variability in scanners, parameters, and analytical pipelines can lead to inconsistent biomarker values across institutions and limit reproducibility, especially for high-dimensional features such as radiomic signatures derived from CT, PET, or MRI [165,166,167,168]. Achieving generalizability in complex modalities such as MRSI and fMRI is even more challenging due to high sensitivity to acquisition parameters and physiological noise. Unlike standard CT, these functional techniques suffer from significant inter-scanner and inter-study variability. Addressing this requires the implementation of standardized phantom-based quality assurance and calibration, as well as advanced post-processing “harmonization” algorithms designed to mitigate noise while preserving biological signals. Initiatives such as the Image Biomarker Standardization Initiative (IBSI) aim to harmonize feature definitions and preprocessing workflows, enhancing cross-software agreement and facilitating multicenter validation of quantitative biomarkers across modalities and scales [169]. However, adoption remains uneven and standards are still evolving, particularly for deep-learning–derived features that capture complex phenotypes beyond handcrafted radiomics definitions. Furthermore, robust validation must move beyond single-center cohorts to ensure these advanced biomarkers remain reliable across different clinical environments.

The current evidence base for imaging biomarkers remains significantly fragmented, with much of the literature limited to single-institution studies and non-standardized endpoints. This landscape further justifies our narrative synthesis as a means to bridge these gaps, highlighting the need for the field to move toward the more uniform data reporting required for future high-level systematic reviews and meta-analyses.

### 5.2. Validation and Clinical Evidence

Most imaging biomarker studies remain retrospective, single-center, and under-powered, with inadequate external validation. Limited sample sizes and heterogeneity in datasets increase the risk of overfitting and false discovery and hinder generalizability to broader populations [170]. Multicenter cohorts with diverse patient populations, prospective validation, and standardized quality assurance frameworks are needed to demonstrate robust associations between biomarker signals, underlying biology, and clinical outcomes. Such validation is especially important for multimodal biomarkers that integrate anatomical, functional, and molecular data, where disparate sources of variability can compound [171]. A significant hurdle in validating imaging biomarkers is the inherent variability of tissue-based ground truths, such as biopsies, which are susceptible to sampling bias and inter-observer variability. If the “gold standard” itself is flawed, it can confound the assessment of imaging-based metrics [172]. To mitigate this, future validation strategies should employ “co-localized” biopsies where imaging coordinates are used to guide tissue sampling, and move toward multi-omics validation that integrates histology with molecular sequencing to provide a more stable and comprehensive ground truth.

### 5.3. Explainability and Interpretability

As AI and machine learning models become central to multimodal biomarker integration, their interpretability and explainability emerge as crucial challenges [173]. Deep learning approaches may offer high predictive performance but are often perceived as “black boxes,” limiting clinician trust and adoption [171]. Efforts to integrate interpretable models or visualization methods (e.g., concept bottleneck models, salient maps) and to link model outputs explicitly to biological features can help bridge this gap, particularly when biomarkers are intended to reflect tumor biology such as hypoxia, perfusion, or cellularity [174,175,176].

### 5.4. Regulatory, Ethical, and Privacy Issues

Imaging biomarkers and associated AI tools must navigate complex regulatory landscapes, including evolving Food and Drug Administration (FDA) frameworks for software as a medical device (SaMD). Demonstrating safety, efficacy, calibration, and traceability across populations is demanding, and existing pathways remain incomplete for AI-driven predictive models. In parallel, ethical concerns including bias, fairness, transparency, and accountability are prominent, particularly when training datasets lack diversity or when models inadvertently perpetuate health disparities. Patient privacy and data security, governed by regulations such as Health Insurance Portability and Accountability Act (HIPAA) or General Data Protection Regulation (GDPR), further complicate large-scale data sharing and collaborative biomarker research, though privacy-preserving technologies such as federated learning show promise.

### 5.5. Data Sharing, Reimbursement, and Equity

The scarcity of large, annotated, multimodal imaging datasets restricts progress, especially in rare tumor subtypes and underrepresented populations. Harmonized data repositories and international consortia are needed to support robust model training and validation and to capture biological diversity across scales. Moreover, the collection of paired high-end imaging and biological/genomic data remains challenging, further contributing to the limited availability of many advanced modalities discussed in this review. At the same time, reimbursement policies for imaging biomarkers and AI tools are poorly defined in many healthcare systems, creating financial disincentives for adoption even when clinical value is evident. Ensuring equitable access to advanced imaging and analytic resources, particularly in low- and middle-income settings, remains a pressing challenge.

### 5.6. Emerging Trends and a Roadmap

Looking ahead, trends that may accelerate clinical translation include tighter integration of imaging biomarkers with biological and genomic data, multiscale modeling frameworks, standardized prospective trials powered for biomarker validation, and improved transparency through open science and shared infrastructures [160]. Harmonization efforts such as IBSI for radiomics and QIBA profiles for quantitative imaging variables are cornerstones for advancing reproducible biomarkers. Continued convergence of technical standardization, regulatory clarity, ethical safeguards, and equitable implementation will be essential to realize the promise of imaging biomarkers in biologically adaptive radiotherapy and personalized oncology.

**Figure 4:**
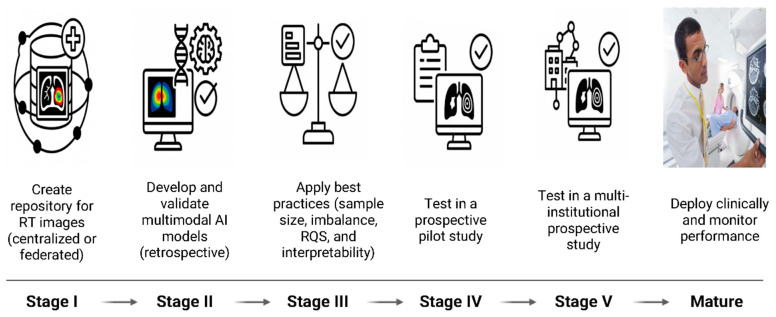
Roadmap for imaging biomarkers in radiotherapy. RT: radiotherapy. AI: artificial intelligence. RQS: radiomics quality score.

To integrate these challenges and efforts into a coherent translational framework, Figure 4 summarizes a multi-stage roadmap for the development, validation, and clinical deployment of imaging biomarkers in radiotherapy.

This multi-stage framework reflects the necessary translational pathway required to move imaging biomarkers from biomarker discovery into routine clinical use, while explicitly addressing technical, biological, and regulatory hurdles at each phase.

At Stage I, the creation of large, well-curated radiotherapy imaging repositories is foundational. This stage directly addresses current limitations in dataset size, diversity, and equity, and enables linkage of imaging biomarkers with clinical, dosimetric, and biological data across institutions. Federated/collaborative learning approaches are particularly attractive for mitigating privacy and regulatory constraints while promoting inclusive data representation [177,178].

Stage II focuses on retrospective development and validation of multimodal AI models that integrate anatomical, functional, and molecular imaging biomarkers across scales. At this stage, biological plausibility and multimodal complementarity are critical, ensuring that imaging features reflect underlying tumor and normal tissue biology rather than spurious correlations. To overcome the scarcity of specialized datasets, the field is moving toward federated learning, which allows for model training across institutions without direct data sharing [177,178]. Additionally, techniques such as transfer learning can leverage models pre-trained on larger, general datasets [179]. Furthermore, synthetic data generation using generative adversarial networks could also be explored to augment small, paired imaging-genomic cohorts.

In Stage III, best practices in model development are emphasized such as the radiomics score and the TRIPOD-AI, including appropriate sample size considerations, handling of class imbalance, and incorporation of interpretability and explainability methods [180,181]. This stage aligns with growing regulatory and ethical expectations for transparency, bias mitigation, and clinical trust, especially for AI-driven imaging biomarkers intended to influence treatment decisions [182].

Stages IV and V represent the transition from technical validation to clinical evidence generation, progressing from prospective pilot studies to multi-institutional prospective trials [183]. These stages are essential for demonstrating reproducibility, generalizability, and clinical utility across diverse patient populations, scanners, and workflows. Importantly, they provide the evidence base needed for regulatory approval, guideline inclusion, and reimbursement considerations.

Finally, the mature deployment phase emphasizes real-world clinical implementation with continuous performance monitoring, recalibration, and post-deployment validation [184]. This reflects an emerging recognition that imaging biomarkers, especially those driven by AI and adaptive radiotherapy platforms, must evolve alongside imaging technology, treatment techniques, and patient populations.

Together, this roadmap highlights that successful translation of imaging biomarkers in radiotherapy requires not only technical innovation, but also rigorous validation, biological grounding, ethical oversight, and equitable implementation. By aligning multimodal imaging, AI, and adaptive radiotherapy within a structured translational framework, the field is moving toward more robust, trustworthy, and clinically impactful biomarker-driven personalization of cancer treatment.

## 6. Conclusions

Imaging biomarkers are rapidly reshaping the role of imaging in radiotherapy, evolving it from a purely anatomical tool to a quantitative and biologically informative framework that supports personalized and adaptive treatment. Advances in MRI, PET, CT/CBCT, and beyond, together with complementary innovations such as radiomics, artificial intelligence, online adaptive radiotherapy, and theranostics, have enabled noninvasive assessment of tumor and normal tissue biology across spatial and temporal scales. Across multiple disease sites, growing clinical evidence demonstrates that imaging biomarkers can refine target delineation, guide dose painting and functional avoidance, and support response assessment and treatment adaptation.

Despite this progress, widespread clinical integration remains constrained by challenges in standardization, reproducibility, validation, interpretability, and regulatory and ethical oversight. Addressing these barriers will require harmonized imaging and analysis pipelines, robust multicenter prospective validation, biologically grounded and interpretable models, and supportive regulatory and reimbursement frameworks. Looking forward, the integration of multimodal and longitudinal imaging biomarkers within adaptive radiotherapy platforms represents a key pathway toward biologically guided, precision radiotherapy. Continued interdisciplinary collaboration and equitable implementation will be essential to translate the promise of imaging biomarkers into routine clinical practice and improved patient outcomes.

## Figures and Tables

**Table 1:** Clinical impact of imaging biomarker-guided radiotherapy. Summary of key clinical trials and landmark studies demonstrating the efficacy of imaging biomarker-guided approaches compared to standard anatomical planning.

Disease Site	Imaging Biomarker-Guided Approach	Outcome vs. Standard Treatment	Key Trials and References
**Brain/** **Glioblastoma**	FET-PET/MRSI-defined biological targeting	Identified infiltration beyond MRI in 65% of cases; reduced marginal recurrence.	SPECTRO GLIO [26]
**Head & Neck**	^18^F-FMISO/^18^F-FAZA PET-guided hypoxic dose painting	Technically feasible dose escalation to 84 Gy; significantly increased TCP.	[43,99,100,101,102]
**Lung**	4DCT ventilation-guided functional lung avoidance	Favorable patient-reported quality of life.	[63]
	PET-adapted dose escalation	~15% Grade 2+ pneumonitis (favorable vs. historical controls).	RTOG 1106 [112]
**Prostate**	mpMRI (PI-RADS)-guided focal boost	Improved biochemical control without increasing late toxicity.	FLAME; hypo-FLAME [130,131]
	PMSA-PET-guided salvage RT	Improved staging and detection; altered management in ~30% of cases.	EMPIRE-1 [133]
**Pancreas**	MR-Linac-guide daily adaptive SBRT	High local control (80–90%) with low Grade 3 GI toxicity (~5%).	SMART Trial [143]
**Rectal**	DWI(ADC)-guided response-adaptive selection	Enabled organ preservation (Wait-and-Watch) in pCR patients.	OPRA Trial [148,149]

## Data Availability

No new data were created or analyzed in this study.

## Abbreviations

4DCT	Four-dimensional Computed Tomography
ADC	Apparent Diffusion Coefficient
AI	Artificial Intelligence
BgRT	Biology-guided Radiotherapy
BOLD	Blood Oxygenation Level-Dependent
BTV	Biological Target Volume
CBCT	Cone-beam Computed Tomography
CT	Computed Tomography
DCE	Dynamic Contrast-Enhanced
DECT	Dual-energy Computed Tomography
DIL	Dominant Intraprostatic Lesion
DL	Deep Learning
DTI	Diffusion Tensor Imaging
DWI	Diffusion-weighted Imaging
FDG	Fluorodeoxyglucose
fMRI	Functional Magnetic Resonance Imaging
FMISO	18F-fluoromisonidazole
GBM	Glioblastoma Multiforme
GDPR	General Data Protection Regulation
HIPAA	Health Insurance Portability and Accountability Act
HNSCC	Head and Neck Squamous Cell Carcinoma
HPV	Human Papillomavirus
IBSI	Image Biomarker Standardization Initiative
IGART	Image Guided Adaptive Radiotherapy
IGRT	Image Guided Radiotherapy
IMRT	Intensity-Modulated Radiotherapy
LLM	Large Language Model
mCRPC	Metastatic Castration-Resistant Prostate Cancer
MRI	Magnetic Resonance Imaging
MRSI	Magnetic Resonance Spectroscopic Imaging
NSCLC	Non-small Cell Lung Cancer
oART	Online Adaptive Radiotherapy
pCR	Pathological Complete Response
PCCT	Photon-counting Computed Tomography
PET	Positron Emission Tomography
PSMA	Prostate-specific Membrane Antigen
PWI	Perfusion-weighted Imaging
QIBA	Quantitative Imaging Biomarkers Alliance
QOL	Quality of Life
RQS	Radiomics Quality Score
RT	Radiotherapy
SaMD	Software as a Medical Device
SBRT	Stereotactic Body Radiotherapy
SPECT	Single-Photon Emission Computed Tomography
SUV	Standardized Uptake Value
TOLD	Tissue Oxygen Level Dependent
